# Item

**Published:** 1898-11

**Authors:** 


### ITEM.

THE JOURNAL has received the following interesting communication:

THOMAS ARMSTRONG, Publisher,
People's Institute Building, Van Buren and Leavitte Streets.

CHICAGO, October 6, 1898.

*Dear Sir*—Will you please send us your advertising rates also state the amount of your paid circulation, and the number of sample copies you send out each issue? We would like to know your lowest rate for carrying the within "ad." one, three, six or twelve months, also what you would charge for a trial insertion. You can see the "ad." in print in the *Medical Brief* of St. Louis, on page 1543, October number; it occupies just one inch. You can state how much the "ad." would cost either with or without your journal for the time mentioned. If we accept your proposition we will return you the "ad." with remittance. You may also state the rates for one inch, or for two inches, and whether your publication is a weekly, semi-monthly or monthly.

Respectfuly yours,

THOMAS ARMSTRONG.

The advertisement referred to and which we were asked to print reads as follows :

JUSTICE TO SUCCESSFUL PRACTITIONERS AND STUDENTS.

Undergraduate practitioners furnishing sworn statements from county officers, certifying they have practised medicine successfully for years, can have the degree of M. D. lawfully conferred at home, without attendance, from legally chartered medical college.

Students attending, graduated when competent, independent of time. Graduation in dentistry same basis. For particulars address, Lock Box 590, Chicago.

The foregoing is respectfully referred to the Illinois State Board of Health for investigation and action. It is difficult to understand how such nefarious schemes can be tolerated. As the JOURNAL does not exchange with the *Medical Brief* we have not seen the advertisement in print in that so-called medical journal, but if the *Brief* has any subscribers in this state we congratulate them on their outlay and the value they get for their money.

